# Colonization potential to reconstitute a microbe community in patients detected early after fecal microbe transplant for recurrent *C. difficile*

**DOI:** 10.1186/s12866-015-0622-2

**Published:** 2016-01-13

**Authors:** Ranjit Kumar, Craig L. Maynard, Peter Eipers, Kelly T. Goldsmith, Travis Ptacek, J. Aaron Grubbs, Paula Dixon, Donna Howard, David K. Crossman, Michael R. Crowley, William H. Benjamin, Elliot J. Lefkowitz, Casey T. Weaver, J. Martin Rodriguez, Casey D. Morrow

**Affiliations:** Center for Clinical and Translational Sciences, University of Alabama at Birmingham, Birmingham, AL 35294 USA; Department of Pathology, University of Alabama at Birmingham, Birmingham, AL 35294 USA; Department of Cell, Developmental and Integrative Biology, University of Alabama at Birmingham, 1918 University Blvd. MCLM 680, Birmingham, AL 35294 USA; Department of Genetics and Heflin Center for Genomic Science, University of Alabama at Birmingham, Birmingham, AL 35294 USA; Department of Microbiology, University of Alabama at Birmingham, Birmingham, AL 35294 USA; Division of Infectious Disease, Department of Medicine, University of Alabama at Birmingham, Birmingham, AL 35294 USA

**Keywords:** Fecal microbiota transplant, *Clostridium difficile*, Niche, Gnotobiotic, Commensal

## Abstract

**Background:**

Fecal microbiota transplants (FMT) are an effective treatment for patients with gut microbe dysbiosis suffering from recurrent *C. difficile* infections. To further understand how FMT reconstitutes the patient’s gut commensal microbiota, we have analyzed the colonization potential of the donor, recipient and recipient post transplant fecal samples using transplantation in gnotobiotic mice.

**Results:**

A total of nine samples from three human donors, recipient’s pre and post FMT were transplanted into gnotobiotic mice. Microbiome analysis of three donor fecal samples revealed the presence of a high relative abundance of commensal microbes from the family *Bacteriodaceae* and *Lachnospiraceae* that were almost absent in the three recipient pre FMT fecal samples (<0.01 %). The microbe composition in gnotobiotic mice transplanted with the donor fecal samples was similar to the human samples. The recipient samples contained *Enterobacteriaceae*, *Lactobacillaceae*, *Enterococcaceae* in relative abundance of 43, 11, 8 %, respectively. However*,* gnotobiotic mice transplanted with the recipient fecal samples had an average relative abundance of unclassified *Clostridiales* of 55 %, approximately 7000 times the abundance in the recipient fecal samples prior to transplant. Microbiome analysis of fecal samples from the three patients early (2–4 weeks) after FMT revealed a microbe composition with the relative abundance of both *Bacteriodaceae* and *Lachnospiraceae* that was approximately 7 % of that of the donor. In contrast, gnotobioitc mice transplanted with the fecal samples obtained from the three at early times post FMT revealed increases in the relative abundance of *Bacteriodaceae* and *Lachnospiraceae* microbe compositions to levels similar to the donor fecal samples. Furthermore, the unclassified *Clostridiales* in the recipient samples post FMT was reduced to an average of 10 %.

**Conclusion:**

We have used transplantation into gnotobiotic mice to evaluate the colonization potential of microbiota in FMT patients early after transplant. The commensal microbes present at early times post FMT out competed non-commensal microbes (e.g. such as unclassified *Clostridiales*) for niche space. The selective advantage of these commensal microbes to occupy niches in the gastrointestinal tract helps to explain the success of FMT to reconstitute the gut microbe community of patients with recurrent *C. difficile* infections.

**Electronic supplementary material:**

The online version of this article (doi:10.1186/s12866-015-0622-2) contains supplementary material, which is available to authorized users.

## Background

*Clostridium difficile* infections are the major causative agent for infective antibiotic associated diarrhea [1, 2]. Infections are most commonly acquired in healthcare settings although community acquired infections are increasingly being reported [1]. In addition to recent use of antibiotics, other risk factors include old age, use of gastric acid suppressing drugs and underlying chronic disease including inflammatory bowel disease [1]. The numbers of infections have been rising during the last decade with estimated health care costs in the billions [3, 4].

The standard treatments for *C. difficile* infection consist of metronidazole, vancomycin, or fidaxomicin, which results in a rate of recurrence at about 20 %; after a third recurrence, the risk of further episodes is even higher [5–7]. Fecal microbiota transplantation (FMT) for treatment of recurrent *C. difficile* has had remarkable success rates for alleviation of the symptoms and restoration of health [8–12].

The reason why FMT is so effective in restoring a microbiome in the gastrointestinal tract of the patients is unknown. Presumably, an effective long-term stable reconstruction would require the commensal microbes in the FMT to access and occupy the niches in the gastrointestinal space following transplantation [13]. Although there have been numerous reports on the composition of the patients microbiota following FMT, there have been no studies to examine the potential of the donor microbes to colonize the recipients post transplant. To gain insights into this issue, we have examined this aspect of microbiome reconstruction following FMT by transplanting human fecal samples into gnotobiotic mice. Since these mice are devoid of microbes, previous studies have shown that the unoccupied (open) niches in the gastrointestinal tract readily accept fecal transplantation and recapitulate the major elements of the human microbiome in the gnotobiotic mice [14, 15]. From the analysis of fecal samples from gnotobiotic mice with transplanted with donor, recipient and post FMT samples, we demonstrate that the microbiota of recipient early post FMT possess the capacity to reconstitute gnotobiotic mice with a microbiome community that is similar to the donor.

## Results

Three patients that had undergone fecal transplants were chosen for this study. All of the three recipients were positive for *C. difficile* at least once and had undergone several rounds of antibiotic treatments without complete resolution of the repeated episodes of colitis. The characteristics of the recipients with respect to age, antibiotic treatment and comorbidities in addition to the *C. difficile* infection can be found in the Additional file 1: Data Set S1. Each recipient agreed to a fecal transplant with individual donors, usually a spouse or other family member. The transplants were accomplished by nasogastric administration. The details for preparation of the donor sample and administration can be found in Additional file 2: Text S1. For all three transplants, fecal samples were collected from donor, recipient and recipient post transplant (collected 2–4 weeks post fecal transplant). By convention, for a given specific transplant (e.g. transplant number 1) we refer to the donor (D), recipient (R) and Recipient post Transplant (RpT) with a prefix letter and the specific transplant number (e.g. D1, R1, RpT1). The Recipient post Transplant samples also carries a suffix which represent time post transplant i.e. RpT1w2 means that the sample is taken 2 week post fecal transplant. The human samples when transplanted in mouse carries a prefix “M” (for example when human sample RpT1w2 is transplanted in mouse it is named MRpT1w2). The list of all human and mouse samples used for microbiota analysis is presented in Additional file 1: Data Set S1.

A total of 9 samples from three human donors, recipients and RpT were collected and transplanted into gnotobiotic mice each as three replicates (27 mouse samples). A total of 36 samples were sequenced with an average sequence depth of 95,464 reads per sample (range 58,972–172877 reads per sample). After quality filtering the sample depth across all samples was normalized to 49,243 reads per sample and used for analysis. A total of 469 operational taxonomic unit (OTU) were identified.

### Comparison of donor, recipient and RpT microbe communities

**Fig. 1:**
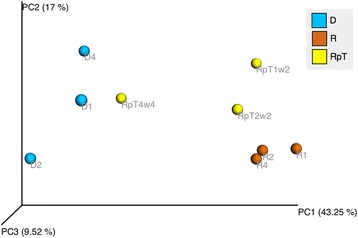
Comparison of the microbial diversity of human donors and recipients pre and post FMT. Principal Coordinate analysis is used to generate 3D PCoA plot (using weighted UniFrac distance metrics) for the fecal samples from the donor (D) and recipient (R) and post FMT (RpT)

**Fig. 2:**
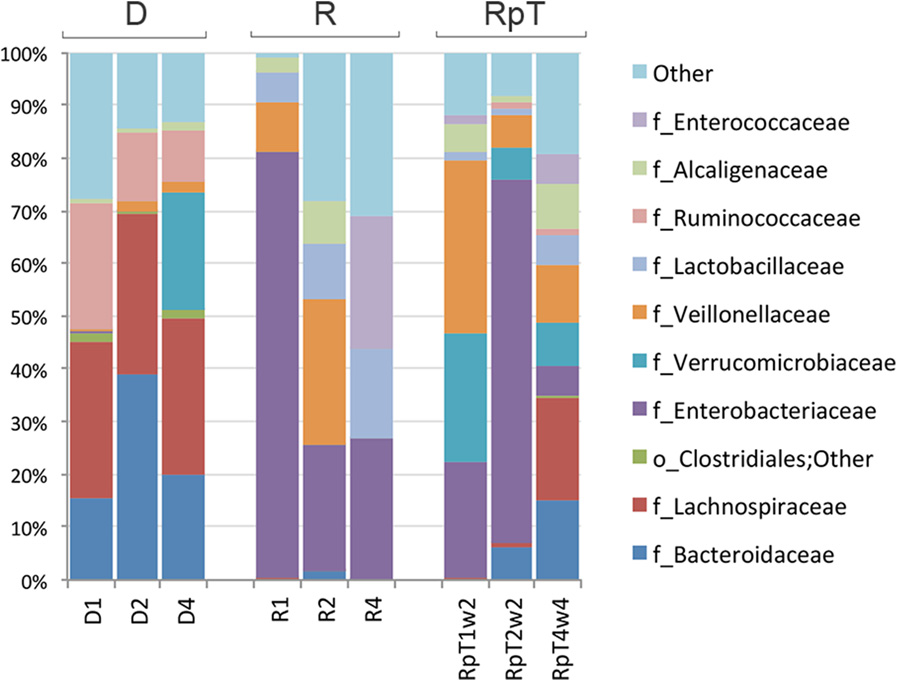
Taxa distribution of human donors and recipients pre and post transplant. A stacked bar plot depicting the taxa distribution at the *family* level of the fecal samples of individual samples from the donors and recipient pre and post FMT

Comparison of the microbe communities from recipient and donor samples using principle coordinate analysis (PCoA) generated from weighted UniFrac metrics revealed each formed separate clusters (Fig. 1). The microbe communities from the post FMT patients (RpT) generally clustered in between the donor or recipient samples on the PCoA plot. The donor samples showed a high abundance of commensal microbes from the families *Bacteriodaceae* (mean 25 %) and *Lachnospiraceae* (mean 30 %). The *Bacteriodaceae* and *Lachnospiraceae* were almost absent in recipient samples (<0.01 % of the abundance of the donors). The recipient samples contained *Enterobacteriaceae*, *Lactobacillaceae*, *Enterococcaceae* in relative abundance of 43, 11, 8 % respectively (these microbes were less that 0.01 % in the donors) (Fig. 2. Additional file 3: Data Set S2). Following FMT, the RpT samples from all three patients at 2–4 weeks showed an increase in the proportional abundance of both *Bacteriodaceae* and *Lachnospiraceae* to approximately 7 %. In addition, the RpT samples showed a decrease in the abundance of families *Enterobacteriaceae*, *Lactobacillaceae*, *Enterococcaceae.* The Shannon’s diversity metric of the recipients (mean 2.63 ± 0.95) was also found to be significantly lower than the donor population (mean 5.18 ± 0.62) (*p* <0.05). The RpT samples show increased diversity (mean 3.42 ± 0.97) when compared with the recipients (Additional file 4: Data Set S3). Collectively, our analysis demonstrates that the microbe compositions of the donor and recipient pre FMT are clearly different. Most importantly, the microbe composition 2–4 weeks after FMT in the three recipients differs from both the donor and pre FMT microbiota.

### Transplant of donor and recipient pre and post FMT microbiota into gnotobiotic mice

It has been previously shown that gnotobiotic mice accept and recapitulate the major elements of the human microbiota after transplant [14, 15]. We wanted to use this feature of gnotobiotic mice to examine the colonization potential of the microbiota from the recipients prior to and after transplant. Following transplant, the mice were housed for 4 weeks for equilibration and the microbiome from fecal samples from each mouse was then analyzed.

**Fig. 3:**
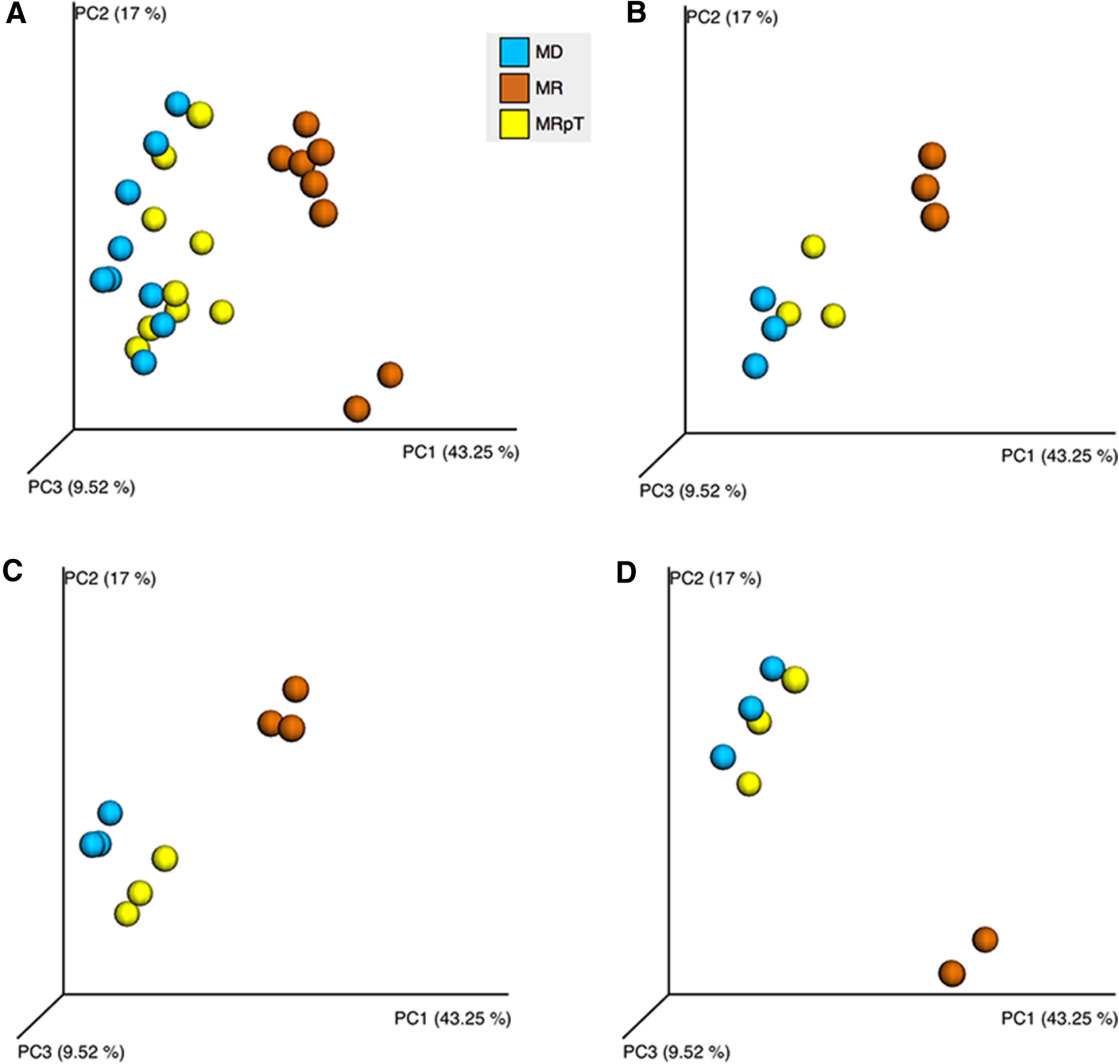
Comparison of the microbiota community in gnotobiotic mice transplanted with fecal samples from donors, recipients pre and post transplant. Gnotobiotic mice were transplanted with the human fecal samples from donor, recipient and RpT samples from FMT 1, 2 and 4. Panel **a** PCoA plot of the fecal samples from all the gnotobiotic mice. The mice transplanted with donor, recipient, and RpT are named as MD, MR and MRpT, respectively. The orange colored spheres are from mice transplanted with the recipient (1, 2 or 4) samples (MR), the blue spheres are from mice transplanted with donor (1, 2 or 4) (MD) and the yellow spheres are mice transplanted with fecal samples of the FMT (MRpT). Panel **b**, **c** and **d** Individual PCoA plots of donor, recipient and RpT from set 1 (Panel **b**), 2 (Panel **c**) and 4 (Pane **d**) transplanted into gnotobiotic mice. Each panel represents the PCoA plot of individual mice transplanted with the donor and recipient pre and post FMT (RpT)

Similar to what we observed from the analysis of the microbiome composition of the donor and recipient pre FMT, the beta diversity comparison using PCoA plots of the weighted UniFrac analysis showed separate clustering of mouse transplanted donor (MD) from the mouse transplanted recipient (MR) (Fig. 3a). The microbiota of MD samples was found to be statistically different (*p* <= 0.001) than the MR samples. The pattern was consistent with all three cases (Fig. 3b, c and d). The transplantation of the fecal samples from RpT from all three of the FMT patients into gnotobiotic mice (MRpT) resulted in a microbe composition in the mice that clustered with the donor (MD): no statistical difference between MD and MRpT samples was found (*p* >0.05).

**Fig. 4:**
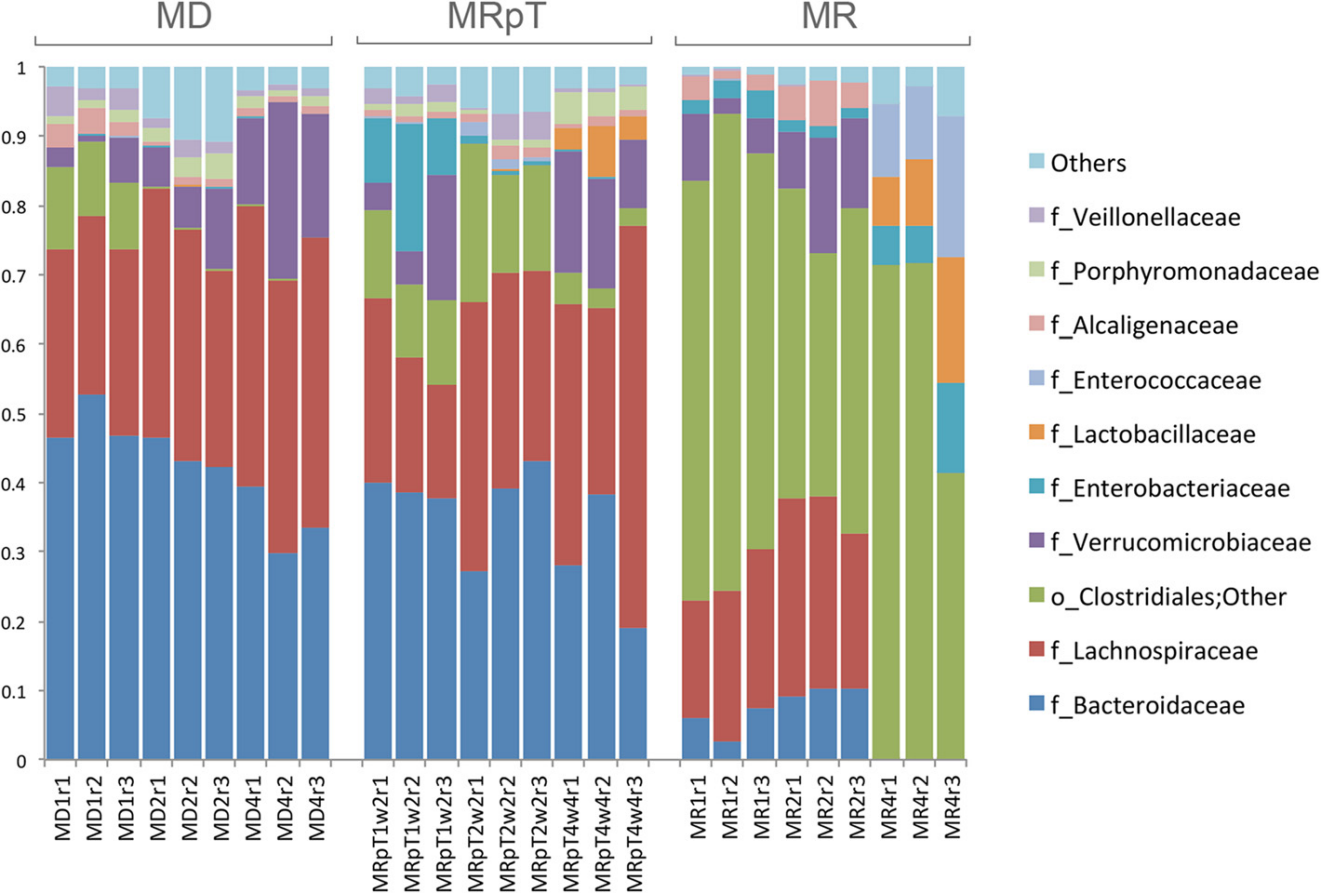
Taxa distribution of the microbial composition of mouse transplanted with donor, recipient pre and post FMT. A stacked bar plot depicting the taxa distribution at the *family* level of the fecal samples of individual gnotobiotic mice transplanted with samples from the donors and recipient pre and post FMT

**Fig. 5:**
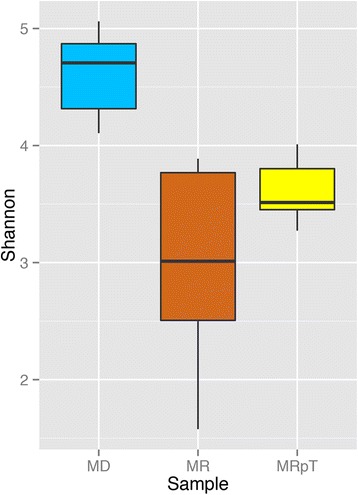
Shannon diversity for gnotobiotic mice transplanted with donor, recipient pre and post FMT. Alpha diversity (Shannon’s diversity) for gnotobiotic mice transplanted with the donor (*blue*), recipients pre (*orange*) and post FMT (*yellow*) microbiota presented as a box plot

The taxa analysis substantiated the microbe clustering seen on the PCoA plots. In particular, the composition of the MRpT from each of the samples from the mice was similar to the composition of the MD with respect to the abundance of the predominant families of commensal microbiota *Bacteroidaceae* and *Lachnospiraceae* (Fig. 4). In contrast, transplantation of the recipient samples into gnotobiotic mice resulted in a microbe composition with an abnormally higher proportion of unclassified *Clostridiales* (referred to as *Clostridiales; Other* which includes all *Clostridiales* which cannot be classified at family level, Additional file 3: Data Set 2) at an average abundance of 55 %. This relative abundance is approximately 7000 times increased from the relative abundance in the recipient samples (i.e. pre-transplant into gnotobiotic mice). Thus, the microbes within the unclassified *Clostridiales* in the recipients have the capacity to colonize and amplify following transplantation into gnotobiotic mice. Further examination of the sequences from the unclassified *Clostridiales* revealed two major OTUs. One OTU (denovo6164) shows 100 % similarity to *C. difficile* (accession number gi:822490352) while the second OTU (denovo14687) shows 100 % similarity to Bacterium NLAE (accession number gi:379364487). Consistent with the increased abundance of the unclassified *Clostridiales* in the MR samples, there was a lower Shannon’s diversity (2.8) as compared to MD (4.6). The Shannon metric of diversity of the MRpT samples was higher than that of the MR samples and similar to that of the MD (Fig. 5).

**Fig. 6:**
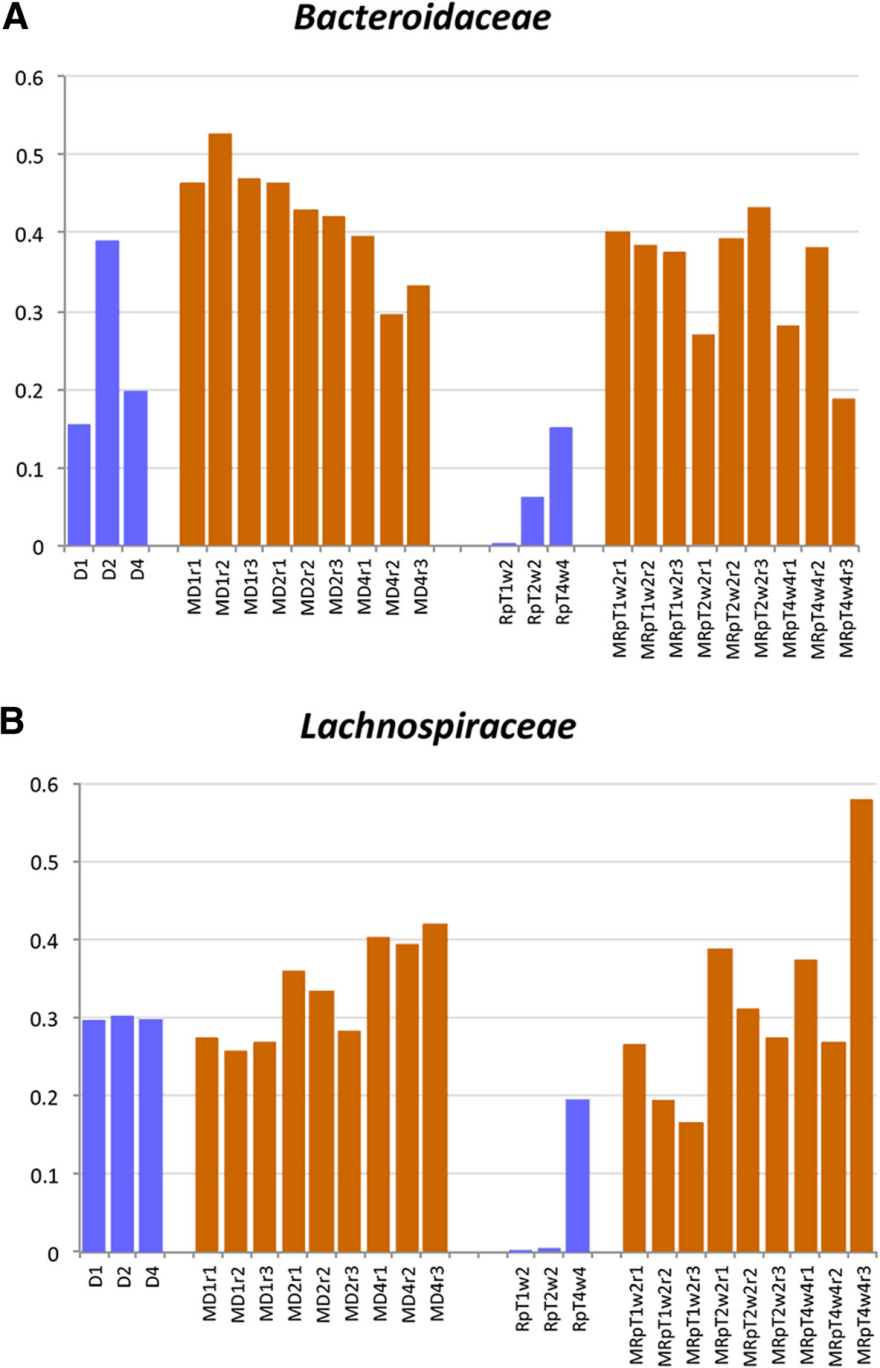
Comparison of relative abundance of selected taxa (family level). The human samples are colored in blue and the gntobiotic mice samples are colored orange. Panel **a** The relative abundance of *Bacteroides* in the human donors and post FMT and their corresponding transplants in gnotobiotic mice. Panel **b** The relative abundance of *Lachnospiraceae* in the human donors and post FMT and their corresponding transplant in gnotobiotic mice

To further highlight the commensal microbe expansion in the gnotobiotics, we next compared the relative abundance of the *Bacteriodaceae* and *Lachnospiraceae* in the human and mouse transplants. The microbes of the human donor samples demonstrated the capacity of the *Bacteriodaceae* and *Lachnospiraceae* to colonize gnotobiotic mice following transplantation (Fig. 6). Even though the RpT samples had lower abundance of *Bacteriodaceae* and *Lachnospiraceae*, the abundance of both increased following transplant into gnotobiotic mice to a similar abundance as gnotobiotic mice transplanted with the donor samples, highlighting the capacity of both *Bacteriodaceae* and *Lachnospiraceae* to effectively colonize and even expand following transplantation (Additional file 3: Data Set 2). In parallel, we found the relative abundance of microbes of the unclassified *Clostridiales* also increased in the MRpT from the human RpT to an average abundance of 10 % from 0.2 % (Data Set 2). However, this increase was only 50 times greater that that found in the human RpT samples as compared to the 7000 times greater for the transplant of the recipient samples in gnotobiotic mice (i.e. human R to MR).

## Discussion

Previous studies have analyzed the microbiome of donor and FMT recipient pre and post-transplant [16–19]. Similar to the results we found with our three patients, these studies found that before FMT, the microbial composition of the recipient was of low diversity and different from the donor [16, 18–20]. Similar to our observation, Shahinas et al. studied 2-week post-FMT samples and noted 50 % samples show noticeable early reconstitution [17]. It is not surprising in our study then, that early after FMT the microbe composition of the recipients was different from both the donor and the recipient.

One of the early events for a successful transplant is the ability of the transplanted microbiota to access the gastrointestinal niches [21]. Once the donor microbes reach the niches, they must also compete for nutrients and space to expand their numbers. To gain insights into the reconstitution of the microbial community in the FMT patients, we took advantage of the capacity of gnotobiotic animals since these mice are devoid of microbes and all of the niches in the gastrointestinal tract would be open for colonization [22–24].

We first analyzed the microbe communities in mice transplanted with the donor or recipient fecal samples. The microbial composition of the mice transplanted with the donor did not cluster with that of mice transplanted with the recipients, consistent to what we saw from the analysis of the donor and recipient obtained from humans. Furthermore, we did not find a difference in the relative abundance of *Bacteroidaceae* and *Lachnospiraceae* in the human and compared to the gnotobioitic. As evidenced from the analysis of the recipient’s microbiota, the microbe composition was very different from that of the donor with increased relative abundance of families *Enterobacteriaceae*, *Lactobacillaceae*, *Enterococcaceae,* consistent with a dysbiotic microbe composition [4, 25]. Transplantation of the recipient fecal samples into gnotobiotic mice though revealed a different composition from that of the recipient with an increase in the relative abundance of the unclassified *Clostridiales*. Thus, even though the unclassified *Clostridiales* microbes represented a minor percentage of the recipient microbes, they had the capacity to amplify and occupy the vacant niches in the gntobiotic mice. The reason why these microbes were not major constituents of the recipient microbe population is unknown. One possibility is that the unclassified *Clostridiales* might have a selective growth advantage in the mouse gut over the other microbes in the recipient’s sample (i.e. families *Enterobacteriaceae*, *Lactobacillaceae*, *Enterococcaceae*). Additional experiments will be required to address this possibility.

We next characterized the colonization potential of the recipient’s fecal sample at early times post FMT. Even though the microbe composition 2–4 weeks after FMT in the three recipients differs from both the donor and pre FMT microbiota, the transplantation of the RpT fecal samples into gnotobiotic animals resulted in a microbe composition that clustered with the donor. We found the proportion of the commensal microbes of the families *Bacteroidaceae* and *Lachnospiraceae* were increased following transplantation into the gnotobiotic mice to levels similar to that of the mice transplanted with the donor samples. This result suggests that the commensal microbes, represented by families *Bacteroidaceae* and *Lachnospiraceae,* in the RpT transplanted into the gnotobiotic mice with the open niches probably out compete the non-commensal microbes present in the sample after FMT for niche space in the gnotobiotic mice. Support for this conclusion comes from previous studies that have shown commensal gut microbes such as *Bacteroides,* have evolved species-specific physical interactions with the host that mediate stable and resilient gut colonization [25–27].

One of the important functions of the commensal gut microbes is to provide an environment to protect against over growth of pathogenic microbes [4, 25, 28, 29]. Indeed, the use of multiple antibiotic treatments to treat *C. difficile* is thought to stress the regenerative properties of the gut microbiota that can promote conditions conducive for over growth of pathogens leading to the dysbiotic microbe composition. It was clear though from the analysis of the RpT transplants in gnotobiotic mice that the microbes of the families *Bacteroidaceae* and *Lachnospiraceae* had the capacity to reconstruct a microbe community that had the capacity to inhibit the expansion of the unclassified *Clostridiales* still present in the fecal samples. In the MRpT samples the presence of the microbes of the families *Bacteroidaceae* and *Lachnospiraceae* (abundance 7 % of that found in the donor) resulted in 50 fold expansion of unclassified *Clostridiales* as opposed to the 7000 fold expansion on the recipient sample that had reduced amounts of the families *Bacteroidaceae* and *Lachnospiraceae* (less that 0.01 % of the donor). It is not clear whether the reduced expansion of the unclassified *Clostridiales* is due solely to the *Bacteroidaceae* and *Lachnospiraceae* out competing for niche space since from the studies of the recipient transplants in gnotobiotic mice the unclassified *Clostridiales* have the capacity to colonize the gnotobiotic mice. Another possibility is that the mouse diet, which is known to impact the gut microbiome composition, might have favored the further expansion of the *Bacteroidaceae* and *Lachnospiraceae* over the unclassified *Clostridales*. Additional studies will be needed to understand the community dynamics that controls the continued reconstruction of the microbe community at these later times following FMT [4, 30].

## Conclusion

Numerous studies have reported on the remarkable success of FMT for treatment to resolve dysbiotic microbiomes that are a result of extensive antibiotics to treat *C. difficile* infections [4, 8–11, 16, 31, 32]. Our results using transplantation of gnotobiotic mice as a model demonstrates the increased colonization potential of commensal microbes (families *Bacteroidaceae* and *Lachnospiraceae*) compared to microbes found in the dysbiotic recipient microbiota. The increased colonization potential of these commensal microbes for the gastrointestinal tract provides a framework to understand the success of FMT to reconstitute the gut microbe community of patients with recurrent *C. difficile* infections.

## Methods

### Sample collection and processing

Fecal samples were collected from donors and recipients prior to and post FMT. The samples were processed and archived using 10 % glycerol as a cryopreservative as previously described [33, 34]. Consent form was obtained for FMT and microbiome analysis as part of an ongoing University of Alabama at Birmingham Institutional Review Board (UAB IRB) approved study at the University of Alabama at Birmingham.

### Isolation of microbial DNA and creation of 16S V4 amplicon library

Microbial genomic DNA was isolated using the Fecal DNA isolation kit from Zymo Research following the manufacturer’s instructions. Once the sample DNA was prepared, PCR was used with unique bar coded primers to amplify the variable region 4 (V4) region of the 16S rDNA gene to create an amplicon library from individual samples [33, 35] (Additional file 2: Text S1).

### Illumina MiSeq DNA sequencing and bioinformatics

The PCR product was ~255 bases from the V4 segment of the 16S rDNA gene, and we sequenced 251 bases single end reads using Illumina MiSeq [33, 35]. FASTQ conversion of the raw data files was performed following de-multiplexing using MiSeq reporter. Quality assessment of the FASTQ files was performed using FASTQC [36] and then quality filtering was done using the FASTX toolkit [37]. Due to low quality of single base toward the 3′ ends of the read, the last base were trimmed for all reads, making the read length as 250 bases. Any read with an average base quality Q score of <20 and the reads with unknown bases (“N”) were discarded. The remainder of the steps was performed with the Quantitative Insight into Microbial Ecology (QIIME) suite, version 1.8 as described below [35, 38, 39]. Chimeric sequences were filtered using the “identify_chimeric_seqs.py” module of USEARCH [40]. Sequences were grouped into operational taxonomic units (OTUs) using the clustering program UCLUST at a similarity threshold of 97 % [40]. The Ribosomal Database Program (RDP) classifier trained using the Greengenes (v13.8) 16S rRNA database [41] was used to make taxonomic assignments for all OTUs at confidence threshold of 80 % (0.8) [42]. The resulting OTU table included all OTUs, their taxonomic identification, and abundance information. OTUs whose average abundance was less than 0.005 % were filtered out [43]. OTUs were then grouped together to summarize taxon abundance at different hierarchical levels of classification (e.g. phylum, class, order, family, genus, and species). These taxonomy tables were also used to generate stacked column bar charts of taxon abundance using Microsoft Excel software (Microsoft, Seattle, WA). Multiple sequence alignment of OTUs was performed with PyNAST [44]. Alpha diversity (within sample diversity) was calculated using Shannon’s metrics as implemented in QIIME (Additional file 4: Data Set S3) [39]. Beta diversity (between sample diversity) among different samples was measured using weighted UniFrac metrics (distance matrices in Additional file 4: Data Set S3) [45]. Principal coordinates analysis (PCoA) was performed by QIIME to visualize the dissimilarity matrix (beta-diversity) between all the samples. 3D PCoA plots were generated using EMPEROR [46].

### Statistical analysis

Samples were grouped into donor, recipient and transplants for both mouse and human. Differences of taxa abundance and alpha diversity between two groups are measured using unpaired T-test (assuming unequal variance) and considered significant at *p* <0.05. Differences in microbiota between groups are measured using PERMANOVA (weighted UniFrac distance).

### Transplantation of archived fecal samples into gnotobiotic mice

Germ-free C57BL/6 mice (3 mice/group) were colonized with archived fecal samples. The sample was first defrosted on ice and a total of 200ul was delivered via the oral and intra-colonic routes to each mouse. Animals with the same fecal samples were housed in the same isolator. Food was autoclaved mouse chow and supplied *ad libitum*. In preliminary experiments, we determined that 2 weeks post transplant was sufficient to allow re-constitution of an intact microbiome. For the current study, fecal samples were taken at 4 weeks. The pellets were processed for DNA and 16S rDNA microbiome analysis is performed. All mice were bred and maintained and all animal experimentation was approved in accordance with guidelines and approval of the University of Alabama at Birmingham Institutional Animal Care and Use Committee.

## Additional files

Additional file 1:
**Characteristics of donors and patients.** (XLSX 12 kb) Additional file 2:
**Preparation of the donor sample and administration.** (DOCX 100 kb) Additional file 3:
**Relative abundance of microbes at family level taxonomy.** (XLSX 69 kb) Additional file 4:
**Alpha and beta diversity metrics.** (XLSX 25 kb)

## Abbreviations

FMT: fecal microbiota transplant
D: donor
R: recipient
RpT: recipient post transplant
w: week
M: mouse
OTU: operational taxonomic unit
PCoA: principle coordinate analysis
IRB: institutional review board
V4: variable region 4 of the rDNA gene
QIIME: quantitative insight into microbial ecology
RDP: ribosomal database program
