# Supplementary material for: Colonization potential to reconstitute a microbe community in patients detected early after fecal microbe transplant for recurrent C. difficile
Source: BMC Microbiol. 2016 Jan 13;16:5. doi: 10.1186/s12866-015-0622-2 (PMC4711103; doi:10.1186/s12866-015-0622-2)
Supplement: Additional file 2: — Preparation of the donor sample and administration. (DOCX 100 kb) [file 12866_2015_622_MOESM2_ESM.docx]

**Supplementary Methods**

**Preparation of sample for FMT.**

All the Donors were in good health and screened for infections that may be transmitted by this FMT within 7 days of the planned FMT procedure:

- 1. Hepatitis A IgM antibody
  2. Hepatitis B core antibody
  3. Hepatitis B surface antigen
  4. Hepatitis C antibody
  5. HIV 1&2 antibody
  6. RPR
  7. Serology for *H. pylori* (IgG antibody)
  8. Stool for *C. difficile* (LAMP) (1)
  9. Stool for ova & parasites (collecting team must communicate with microbiology laboratory resident so that the test is not automatically cancelled)
  10. Stool for feces culture

The Recipients was tested for:

- 1. Hepatitis B surface antibody quantitative
  2. Hepatitis B surface antigen
  3. Hepatitis C antibody
  4. HIV 1&2 antibody
  5. RPR
  6. Stool for *C. difficile* (LAMP) if not available in the records or in our system.

Once screening was complete the Donor collected a stool sample in a standard closed container used for stool collection. The sample was processed and prepared in a laboratory in the Division of Infectious Diseases as previously described (2).

Recommendations for volumes to be used depending on route of administration:

Table 1

|  | Upper gastrointestinal  tract | Lower gastrointestinal  tract |
| --- | --- | --- |
| Donor stool volume | 25-30 g | 25-30 g |
| Volume of dilution in saline | 50-100 cc | 200-500 cc |

The stool sample was homogenized stool with sterile non-bacteriostatic saline or water in the blender (see Table 1 for volume). Initially use the low setting until the sample breaks up, and then advance the speed gradually to the highest setting and was continued for 2 – 4 minutes until sample was smooth. The suspension was filtered using a 90mm Perforated filter plate sample collected in sterile container with capacity of 100-500 cc depending on the planned route of administration (Table 1).

The procedures were performed by physicians with experience in placing nasogastric tubes, nasoduodenal tubes, nasojejunal tubes, administering enemas, or performing colonoscopies (in case of GI co-investigators). In those who need an NG or ND tube an X ray will be obtained prior to the transplant to make sure the tube is well positioned. After the procedure the patients were asked to follow up in clinic or continue to be seen in the hospital if they are inpatients.

**PCR for V4 region of 16S rDNA.**

The oligonucleotide primers used for the PCR amplification of the V4 region of the 16S rRNA gene were as follows (Eurofind Genomics, Inc., Huntsville, AL):

Forward V4:

5’AATGATACGGCGACCACCGAGATCTACACTATGGTAATTGTGTGCCAGCMGCCGCGGTAA 3’; and

Reverse V4:

5’CAAGAGAAGACGGCATACGAGATNNNNNNAGTCAGTCAGCCGGACTACHVGGGTWTCTAAT3’.

For PCR reactions, the conditions were as follows:

10 µL of 5X Reaction Buffer; 1.5 µL (200 uM) of each of the dNTPs; 2 µL (1.5 uM) of each of the primers; 1.5 µL (5 U) of the “LongAmp” enzyme kit (New England Biolabs, Ipswich, MA; cat # E5200S); 30 µL 2-5 ng/ul of the Template DNA prepared using the “Fecal DNA Isolation kit with the concentration of DNA; 3µL of H2O to a total reaction volume of 50 µL. The PCR cycling parameters were initial denature 94o C 1 min; 32 cycles of amplification in which each cycle consisting of 94oC 30 secs, 50oC 1 min, 65oC 1 min; followed by final extension of 65oC 3 min; then final hold at 4o C. Following PCR, the entire PCR reaction was electrophoresed on a 1.0% (w/v) agarose/Tris-borate-EDTA agarose gel. The PCR product (approximately 380 bp predicted product size) was visualized by UV illumination. The DNA band was excised with a sterile scalpel and purified from the agarose using QIAquick Gel Extraction Kit according to manufacturer’s instructions. (Qiagen; cat # 28704). The samples were quantitated using Pico Green and adjusted to a concentration of 4 nM (3).

**References**

1. Boyanton, B.L., Jr., et al., *Loop-mediated isothermal amplification compared to real-time PCR and enzyme immunoassay for toxigenic Clostridium difficile detection.* J Clin Microbiol, 2012. **50**(3): p. 640-5

2. Landy, J. H. O. Al-Hassi , S. D. McLaughlinà, A. W. Walker, P. J. Ciclitira, R. J. Nicholls*, S. K. Clark and A. L. Hart. 2011. Review article: faecal transplantation therapy for gastrointestinal disease. Aliment Pharmacol Ther; 34: 409–415.

3. Kumar, R., P. Eipers, R.B. Little, M. Crowley, D.K. Crossman, E.J. Lefkowitz and C.D. Morrow. 2014. Getting Started with Microbiome Analysis: Sample Acquisition to Bioinformatics. Current Protocols in Human Genetics. Curr. Protoc. Hum. Genet. 82:18.8:18.8.1–18.8.29 (PMID 25042718).
